# Microbiota and Tuberculosis: A Potential Role of Probiotics, and Postbiotics

**DOI:** 10.3389/fnut.2021.626254

**Published:** 2021-05-07

**Authors:** Yue Liu, Jiaqi Wang, Changxin Wu

**Affiliations:** ^1^Key Laboratory of Medical Molecular Cell Biology of Shanxi Province, Institutes of Biomedical Sciences, Shanxi University, Taiyuan, China; ^2^The Provincial Key Laboratories for Prevention and Treatment of Major Infectious Diseases Shanxi, Institutes of Biomedical Sciences, Shanxi University, Taiyuan, China

**Keywords:** tuberculosis, microbiota, probiotics, postbiotics, immunity

## Abstract

Tuberculosis (TB), caused by *Mycobacterium tuberculosis* attacking the lungs and other organs, is one of the most common infectious disease worldwide. According to the WHO's 2020 report, a quarter of the world's population were infected with *M. tuberculosis*, and ~1.4 million people died of TB. Therefore, TB is a significant public health concern, which requires cost-effective strategies for prevention and treatment. The microbiota has been considered as a “forgotten organ” and a complex dynamic ecosystem, which plays a significant role in many physiological processes, and its dysbiosis is closely associated with infectious disease. Recently, a few studies have indicated associations between TB and microbiota. This review summarizes studies concerning the alterations of the gut and respiratory microbiota in TB, and their relationship with host susceptibility to *M. tuberculosis* infection, indicating that microbiota signatures in different stages in TB progression could be considered as biomarkers for TB diagnosis and control. In addition, the potential role of probiotics and postbiotics in TB treatment was discussed.

## Tuberculosis

**Figure 1:**
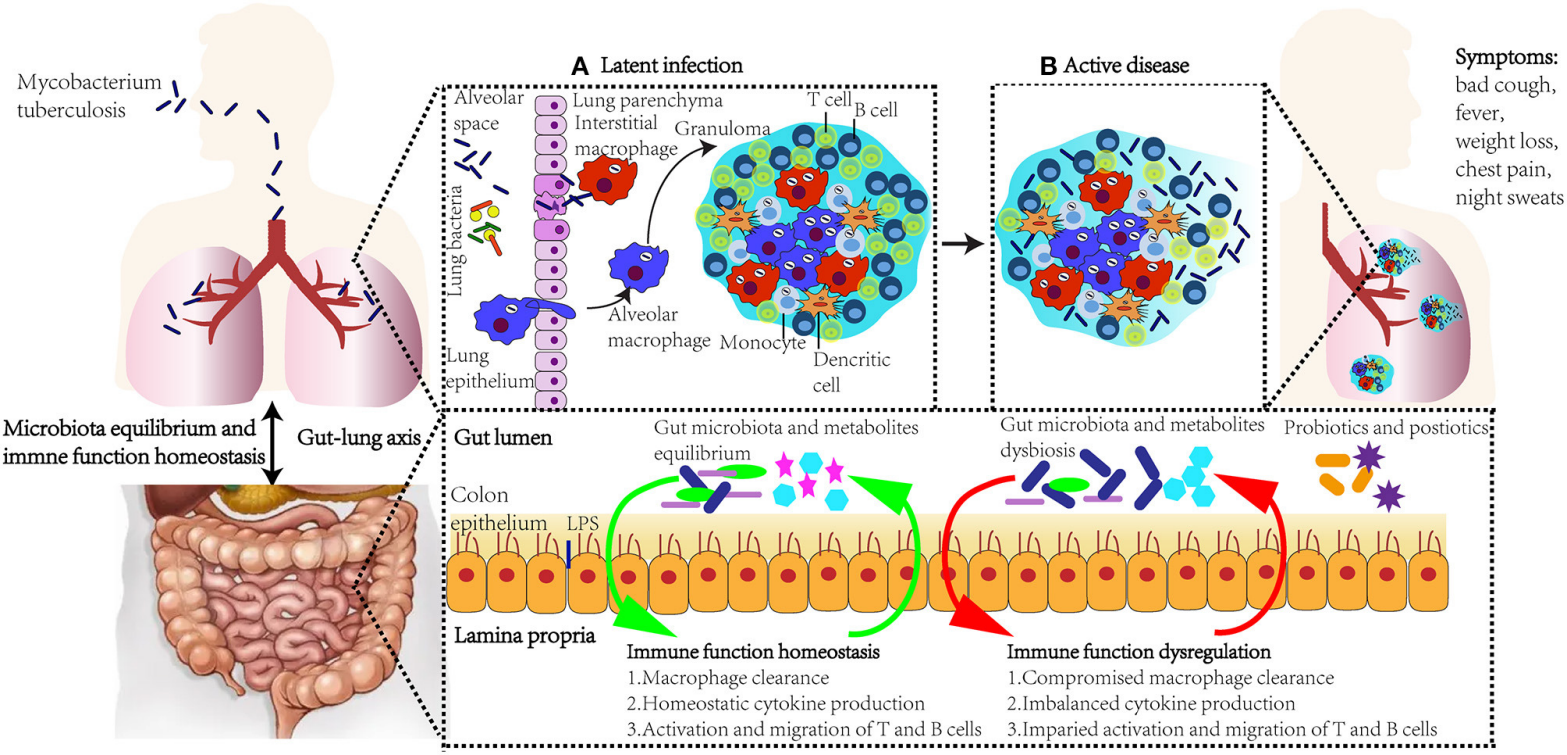
The role of immune response, gut microbiota, lung microbiota, and gut–lung axis in *M. tuberculosis* infection. Upon exposure to *M. tuberculosis*, alveolar epithelial cells are the first cell lines to recognize and bind to the outer surface molecules of the mycobacteria. Immune cells, including macrophages and T and B cells, migrate to infection sites, form granulomas around *M. tuberculosis*, and restrict its replication **(A)**. However, when granulomas are impaired due to factors, such as HIV infection, smoking, aging, and malnutrition, *M. tuberculosis* escape from granulomas and spread to other tissues **(B)**. Active TB patients show symptoms such as bad cough, fever, weight loss, chest pain, and night sweats. The gastrointestinal tract and lung influence the microbiota and immune function homeostasis of each other, and this bidirectional gut-lung axis consequently influences the host immune response against *M. tuberculosis*. Novel supplementation, such as probiotics and postbiotics, could modulate microbiota and regulate immune function, and be applied as strategies in TB prevention and treatment.

Tuberculosis (TB), typically caused by *Mycobacterium tuberculosis* infection, is a highly communicable infectious disease (1). According to the WHO's 2020 report, in 2019, around 10 million people were infected with and developed TB, and 1.4 million deaths occurred (1). *M. tuberculosis* can be expelled by TB patients, spread through the air and infect others (2). Not only the lungs, other organs, such as the brain and spine can also be invaded by *M. tuberculosis* (1). At the onset of *M. tuberculosis* invasion, its cell wall components are recognized by pathogen-recognition receptors (PRRs), consequently activating the innate immune response (3). Antimycobacterial activity of alveolar macrophages is activated by tumor necrosis factor-alpha (TNF-α) and interferon gamma (IFN-γ). Immune cells, including macrophages, neutrophils, and T and B cells, migrate to infection sites, form granulomas around *M. tuberculosis*, and restrict its replication (latent TB infection) (4). At this point, *M. tuberculosis* can still survive and replicate in granulomas by inhibiting the maturation of phagolysosomes and destructing the patterns of cell death and immune response. However, when granulomas are impaired due to factors, such as HIV infection, smoking, aging, and malnutrition, *M. tuberculosis* can escape from granulomas and spread to other tissues (active TB infection) (5). Most individuals with *M. tuberculosis* invasion remain symptom-free (latent TB infection), and 5–10% of the ~2 billion infected people will develop active TB, showing symptoms, such as bad cough, fever, weight loss, chest pain, and night sweats (Figure 1). People with diabetes, alcohol intake disorder, HIV infection, and smoking have a higher probability of developing TB (6). TB typically requires extended treatment with broad spectrum antibiotics for 6-9 months, hence generally resulting in drug-resistant TB (1). Therefore, it is significant to introduce novel strategies to control TB and improve treatment outcome.

## Microbiota and Tuberculosis

The concept of human microbiota referring to “microbial community structure in habitats of the human body such as the skin, vagina, oral cavity, gut, and lower respiratory tract” was firstly introduced by Lederberg and McCray (7). The microbiota has been considered as a “forgotten organ” and a complex dynamic ecosystem, which plays a significant role in many physiological processes, including digestion and nutrient absorption, and regulation of the immune system (8). The gut microbiota equilibrium plays a positive role in systemic and lung immunity, through regulating the differentiation of T cells, migration and apoptosis of immune cells, activation of toll-like receptor signaling, and suppressing inflammatory tone (9). The pulmonary microbiota is also crucial to protect against pathogens, regulate Th1/Th2 immune response, and control inflammation (10). Therefore, human microbiota could play an important role against *M. tuberculosis* infection. Consequently, microbiota alterations may contribute to the spectrum of TB pathogenesis, and it is critical to characterize the human microbiota profile at every stage of *M. tuberculosis* infection, which may be helpful to achieve the goal of the End TB Strategy, introduced by WHO (11). Available studies concerning the gut and pulmonary microbiota in healthy and TB patients, effects of anti-TB treatment on microbiota, and the association between microbiota composition and TB treatment efficiency are summarized in Table 1.

**Table 1:** Example of studies designed to determine the relationship between gut microbiota and tuberculosis.

**Host/source**	**Sample size**	**Location**	**Methods**	**Results**	**References**
Human	13 healthy controls, 28 TB patients, 23 TB patients anti-TB therapy	Shenzhen, China	Fecal samples, 16S rRNA sequencing, V4 region	*M. tuberculosis* infection led to a decreased α diversity, mainly associated with alterations in *Bacteroides* genus. During anti-TB treatment, genus *Clostridiales* significantly decreased, *Bacteroides* OTU230 and *Bacteroides fragilis* increased.	(12)
Human	31 healthy controls, 46 TB patients	Beijing, China	Fecal samples, metagenomic Sequencing	Declined microbiota diversity and number, remarkable decline in SCFAs -producing bacteria. SNPs in the species of *Bacteroides vulgatus* were dramatically different.	(13)
Human	20 healthy controls, 19 NTB, and 18 RTB	Chengdu, China	Fecal samples, 16S rRNA sequencing, V4 region	Actinobacteria and Proteobacteria were significantly higher, Bacteroidetes were lower in the RTB patients compared to controls. *Prevotella* and *Lachnospira* were dramatically lower in NTB and RTB compared to the control group.	(14)
Human	18 healthy children, 18 pediatric TB patients	Sichuan, China	Fecal samples, 16S rRNA sequencing, V3-V4 region	Declined microbiota diversity, increased abundance of *Prevotella, Enterococcus*, and reduced abundance of *Ruminococcaceae, Bifidobacteriaceae*, and *prausnitzii*.	(15)
Human	23 healthy controls, 25 TB patients	Taipei	Fecal samples, 16S rRNA sequencing, V3-V5 region	Decreased ratio of *Firmicutes* to *Bacteroidetes* in TB patients compared to healthy controls.	(16)
Human	16 healthy controls, 25 TB patients	New Delhi, India	Fecal samples, 16S rRNA sequencing, V6-V7 region	Firmicutes and Actinobacteria significantly increased in TB patients.	(17)
Female C57BL/6 mice, 6-7 week	CT: control mice; Abx: Abx treated mice; Mtb: mice infected by *M. tuberculosis*; Abx-Mtb: mice after treated with Abx, mice infected by *M. tuberculosis*; Mtb-INH: mice infected by Mtb and treated with INH; Abx-Mtb-INH: Abx treated mice infected with *M. tuberculosis* prior to INH therapy (n = 5 mice per group or cage).	Punjab, India	Fecal samples, quantitative real-time PCR	Abx led to decreased abundance of commensal bacteria *Campylobacter, Bifidobacterium*, and *Lactobacillus*, and increased abundance of *Enterococcus* and *Bacteroides*.	(18)
Human	52 healthy controls, 6 patients with MDR-TB treatment, 18 patients recovered from MDR-TB treatment	Linyi, China	Fecal samples, 16S rRNA sequencing, V3-V4 region	26% drop in microbiota diversity and significant changes in composition in patients with MDR-TB compared to controls. 16% drop in microbiota diversity and altered taxonomic composition in patients recovered from MDR-TB compared to controls.	(19)
Human	50 healthy controls, 19 TB patients with TB therapy	Port-au-Prince, Haiti	Fecal sample, 16S rDNA and metagenomic DNA sequencing	TB therapy deplete multiple immunologically significant commensal bacteria, such as *Ruminococcus, Eubacterium, Lactobacillus*, and *Bacteroides*, persist for at least 1.2 years.	(20)
Female mice of 4–8-week-old C57BL/6J-CD45a	CT: control mice; TB+HRZ: mice challenged with *M. tuberculosis* followed by HRZ therapy (*n* = 5 mice per group or cage).	Evanston, USA	Fecal sample, 16S rRNA sequencing, V3-V4 region	HRZ could immediately and reproducibly cause microbiota composition changes during the whole treatment course, and even 3 months after the treatment stop, with significant decreases in members of class Clostridia.	(21)
Human	10 healthy controls, 6 TB patients	PCTY Yucatán, Mexico	BAL samples, 16S rRNA sequencing, V3-V4 region	The diversity was decreased in TB patients compared to healthy volunteers, characterized by a significant decline in *Streptococcus* genus and increase in *Mycobacterium*.	(22)
Human	70 healthy controls, 70 TB patients,	Shenzhen, China	BAL samples, 16S rRNA sequencing, V3-V4 region	The α diversity was decreased in TB patients compared to healthy volunteers	(23)
Human	14 healthy controls, 22 TB patients	Hong Kong, China	Sputum sample, 16S rRNA sequencing, V1-V2 region	TB patients were characterized by *Proteobacteria*, and *Bacteroidetes*, while those of healthy controls were *Firmicutes*. In addition, the abundance of *Mogibacterium, Moryella*, and *Oribacterium* was increased significantly in the TB patients compared to controls.	(24)
Human	20 healthy controls, 25 NTB patients, 30 RTB patients, and 20 TB patients with treatment unsuccessful	Shanghai, China	Sputum sample, 16S rRNA sequencing, V1-V2 region	An increase in abundance of *Streptococcus, Gramulicatella* and *Pseudomonas*, and a decrease in abundance of *Prevotella, Leptotrichia, Treponema, Catonella*, and *Coprococcus* in TB patients compared those in the healthy controls.	(25)

*SCFAs, short chain fatty acids; NTB, new tuberculosis patients; RTB, recurrent tuberculosis patients; Abx, broad-spectrum antibiotics; MDR-TB, multi-drug-resistant tuberculosis; BAL, bronchoalveolar lavage; HRZ, isoniazid-rifampin-pyrazinamide*.

### Gut Microbiota Alterations in TB Patients

Gut microbiota plays a significant role in modulating the host immune system (6). Gut microbiota could be significantly changed due to pulmonary infection with influenza virus stemming from a mechanism dependent on type I interferons (26). Several studies have found that there were significant differences between TB patients and healthy controls in gut microbiota. In a cross-sectional research study, Hu et al. (12) characterized the gut microbiota profile in Chinese TB patients and found that *M. tuberculosis* infection led to a decreased α diversity, which was mainly associated with alterations in *Bacteroides* relative abundance. Another research group in China recruited 31 healthy controls and 46 TB patients, and observed significant declined microbiota diversity and number, characterized by a remarkable decline in short chain fatty acid (SCFA)- producing bacteria. Besides, single nucleotide polymorphisms (SNPs) in the species of *Bacteroides vulgatus* were dramatically different in TB patients compared to heathy controls (13). A research group by Luo, Liu (14) divided their TB patients into new tuberculosis patients (NTB) and recurrent tuberculosis patients (RTB). Their results showed that Actinobacteria and Proteobacteria were significantly higher, while Bacteroidetes, containing a variety of beneficial commensal bacteria, were lower in the fecal samples of RTB patients. In addition, *Prevotella* and *Lachnospira* were dramatically lower in the NTB and RTB compared with the healthy control group. Similar results were also found in children with TB. A case-controlled study found that the gut microbiota profile in pediatric TB patients was characterized by declined microbiota diversity, increased abundance of *Prevotella* and *Enterococcus*, and reduced abundance of Ruminococcaceae, Bifidobacteriaceae, and *Faecalibacterium prausnitzii*, which are beneficial to host health (15). Another study found a decreased ratio of *Firmicutes* to *Bacteroidetes* in TB patients compared to healthy controls using 16S rRNA sequencing (16). A research group in India applied 16S rRNA sequencing to distinguish microbiota composition between TB patients and healthy controls, and found that Firmicutes and Actinobacteria were significantly increased in TB patients (17). According to these studies, a distinct gut microbiota profile could be observed between healthy and TB patients, and microbiota signatures in different stages in TB progression could also be identified. However, the causal relationship and whether an altered gut microbial profile with declined bacterial diversity increases the susceptibility to TB needs to be further investigated.

### Effects of Anti-TB Treatment on Gut Microbiota

Antibiotic administration could critically affect gut microbiota, leading to the disruption of bacterial equilibrium (27). Broad-spectrum antibiotics could decrease richness, diversity, and evenness of the whole bacteria community, and after antibiotics, gut microbiota could either recover or achieve a new balance (28). The impact of antibiotics used in TB treatment on gastrointestinal microbiota has also been investigated. Hu et al. (12) found that anti-TB therapy could lead to rapid, dramatic changes in the diversity and composition of the microbiota community. During anti-TB treatment, the relative abundance of genus *Clostridium* significantly decreased, whereas several members of the *Bacteroides* genus, such as *Bacteroides fragilis* and *Bacteroides* OTU230 increased. Besides, after 1 week of TB treatment, OTU8 and OTU2972 assigned to the family Erysipelotrichaceae strikingly increased, whereas the rest of the Erysipelotrichaceae family declined. Similarly, Negi, Pahari (18) applied an *in vivo* mouse model and found that broad-spectrum antibiotics could lead to significant alterations in gut microbiota composition with decreased abundance of commensal bacteria *Campylobacter, Bifidobacterium*, and *Lactobacillus*, and increased abundance of *Enterococcus* and *Bacteroides*. Multiple antibiotics in the case of TB treatment could not only result in immediate dramatic changes in gut microbiota composition but also even after a long period of recovery. Wang, Xiong (19) found that multi-drug-resistant tuberculosis (MDR-TB) treatment could result in changes in gut microbiota with a 26% decline in microbiota diversity and a significant change in microbiota composition. In addition, there was a 16% decrease in gut microbiota community richness in recovered patients from MDR-TB treatment compared to the untreated group. However, this study only recruited six MDT-TB treated volunteers, hence interpretation of these results should be cautious. These results were in agreement with previous studies. Wipperman, Fitzgerald (20) found that TB therapy could dramatically deplete multiple immunologically significant commensal bacteria, such as *Ruminococcus, Eubacterium, Lactobacillus*, and *Bacteroides*. These microbiota alterations could even persist for at least 1.2 years. Using an *M. tuberculosis*-infected mouse model, Namasivayam, Maiga (21) found that isoniazid-rifampin-pyrazinamide treatment could immediately and reproducibly cause microbiota composition changes during the whole treatment course, and even 3 months after the end of treatment, with significant decreases in members of class Clostridia, such as *Acetivibrio, Robinsoniella, Alkaliphilus, Stomatobaculum, Butyricicoccus, Acetanaerobacterium, Tyzzerella, Ruminococcus*, and *Peptococcus*.

### Gut Microbiota Alterations and Anti-TB Treatment Efficiency

Several diseases such as cancer, allergies, autoimmune diseases, and infections, could be triggered and aggravated by altered gut microbiota composition (29, 30), highlighting the significance of gut microbiota in treatment efficiencies in hosts. A few studies have indicated that altered microbiota balance could limit the potency of anti-TB drugs and TB treatment efficiency. Negi et al. (18) applied an *in vivo* mouse model to study the influence of gut microbiota dysbiosis on isoniazid (INH) efficiency against *M. tuberculosis*, and found that a declined abundance of *Lactobacillus, Bifidobacterium*, and *Campylobacter* caused by antibiotic pre-treatment could lead to immune response impairment to INH treatment in *M. tuberculosis* clearance and more serious granulomatous development. In addition, this group also demonstrated that impairment of the intestinal innate defense and immunity stemmed from microbiota changes during INH therapy, and resulted in lower levels of antimicrobial peptide RegIII γ and pro-inflammatory cytokines TNF-α and IFN-γ, and higher levels of anti-inflammatory cytokine IL-10. Khan, Mendonca (31) also found that intestinal changes with increased abundance of *Bacteroides* and *Verrucomicrobiaceae*, and decreased abundance of *Lachnospiraceae* compromised alveolar macrophage immune function to *M. tuberculosis*. Similar results were found previously. Dumas et al. (32) demonstrated that reduced Bacteroidetes and Firmicutes, and increased Proteobacteria of antibiotic pre-treatment mice was associated with an increased early lung colonization by *M. tuberculosis*, and indicated the role of microbiota in contributing to early protection possibly through sustaining the functions of mucosal-associated invariant T cells. These findings suggest a role of gut microbiota in anti-TB treatment. Microbiota alterations could impair anti-TB treatment in *M. tuberculosis* survival and clearance. In addition, gut microbiota could influence the pharmacokinetics of anti-TB drugs, through producing enzymes which can activate or inactivate drugs, and binding to drugs hence influencing bioavailability (33). Therefore, modulating gut microbiota and maintaining equilibrium using probiotics and postbiotics could enhance the efficiency of anti-TB drugs and improve host immunity against *M. tuberculosis*.

### Pulmonary Microbiota Alterations in TB Patients

The taxa of pulmonary microbiota have been demonstrated to be similar to those along the respiratory tract, with a declined microorganism burden compared with those in the oral cavity (34). There is a balance of dynamic bacterial community shift along the respiratory tract, which results from gastric content aspiration, mucosa dispersion and elimination, coughing, and immunity (35). However, if this equilibrium is disrupted, the microbiota community would significantly change and be associated with lung disease (36, 37). A few studies have described the pulmonary microbiota composition in TB patients, some collected bronchoalveolar lavage (BAL) samples, and some collected sputum samples. BAL samples are mainly used to describe microbiota colonized in the lower respiratory tract, although they are difficult to collect and are potentially contaminated by oral microbiota. Vázquez-Pérez et al. (22) used BAL samples to describe and compare the pulmonary microbiota composition in TB patients and healthy volunteers. Using 16S rDNA sequencing methods, the diversity of microbiota was found to be decreased in TB patients compared to healthy volunteers, characterized by a significant decline in *Streptococcus* genus and increase in *Mycobacterium*. Similar results have also been demonstrated in Chinese TB patients by Hu et al. (23), with lower α diversity of pulmonary microbiota composition in BAL in TB patients. Meanwhile, a few studies also investigated the respiratory microbiota profile in sputum samples which are non-invasively and more easily collected compared to BAL samples. After comparing sputum samples from 22 TB patients and 14 healthy controls, Cheung et al. (24) found that the pulmonary microbiota of TB patients were characterized by Proteobacteria and Bacteroidetes, while those of healthy controls harbored Firmicutes. In addition, the abundance of *Mogibacterium, Moryella*, and *Oribacterium* were increased significantly in the TB patients compared to controls. A research group by Wu et al. (25) further divided TB patients into NTB, RTB, and treatment failure TB patients, and compared their sputum samples. They showed an increase in abundance of *Streptococcus, Gramulicatella*, and *Pseudomonas*, and a decrease in abundance of *Prevotella, Leptotrichia, Treponema, Catonella*, and *Coprococcus* in TB patients compared those in the healthy controls. The results also showed that RTB had higher *Pseudomonas /Mycobacterium* and lower *Treponema/Mycobacterium* ratios compared to NTB. Besides, the abundance of *Pseudomonas* and *Pseudomonas*/*Mycobacterium* increased in patients whose TB treatment were unsuccessful compared to NTB. According to these studies, pulmonary microbiota diversity is observed to be decreased in TB patients compared to healthy controls. In addition, the dominant lung microbiota species are different between TB patients and healthy controls. These findings also suggest the important role of pulmonary microbiota in TB pathogenesis and treatment efficiency, consequently more attention should be paid to pulmonary microbiota for improving TB control strategies and treatment efficiencies in the future.

## Potential Role of Probiotics and Postbiotics

As mentioned above, gut microbiota alterations could impair the function of macrophages and disrupt the activation of immune cells in *M. tuberculosis* clearance. Therefore, supplementation which can modulate gut microbiota and maintain equilibrium could be applied to improve host immunity against *M. tuberculosis*, and enhance the treatment outcome of anti-TB drugs.

In the early twentieth century, it was hypothesized by Metchnikoff that the long life span of Bulgarian peasants resulted from their large intake of fermented milk which contained beneficial bacteria, and the term probiotic was initially proposed (38). In 2014, probiotics were stipulated as “defined contents, appropriate viable count at end of shelf life, and suitable evidence for health benefits,” and the safety of probiotics were addressed by Hill (39). Probiotics have been shown to modulate microbiota composition through inhibiting growth and activity of harmful bacteria and pathogens, and stimulating those of beneficial bacteria (40). Furthermore, probiotics can modulate the host immune system through stimulation of host immunoglobulins and antibacterial compounds, and enhancement of the innate and adaptive immune response (41). In an *in vitro* study, probiotic bacteria *Lactobacillus brevis, L. plantarum*, and *L. fermentum* showed antimicrobial activity against *M. tuberculosis* (42). In another *in vitro* study, probiotic *L. casei, L. plantarum*, and *L. salivarius* showed strong antimicrobial activity against *M. bovis* Bacillus Calmette-Guerin (BCG), and this anti-mycobacterial activity may have stemmed from the metabolites produced by the *Lactobacillus* species, which harbor genes encoding for class II bacteriocins and bacteriolysins. Furthermore, *L. plantarum* significantly decreased BCG intake by phagocytes, whereas *L. casei* increased BCG intake and *L. salivarius* had no effect on it (43). The inhibitory activity against *M. tuberculosis* by lactobacilli is in agreement with a previous study (44). In an *in vivo* mouse model, Negi et al. (45) found that a decrease in Bacteroidetes and Firmicutes, and an increase in Proteobacteria caused by antibiotics could result in the declined expression of macrophage-inducible Ca^2+^-dependent lectin receptor (mincle), which functions as a pattern recognition receptor recognizing and binding to the carbohydrate structure on pathogens including those on *M. tuberculosis*, and subsequently induce an innate immune response (46). In addition, gut microbiota alterations lead to increased burden of *M. tuberculosis*, a decreased effector and memory T cell population, and increased regulatory T cells in the lungs (45). However, probiotic supplementation with *Lactobacillus plantarum* MTCC 2621 could restore mincle and MHC-II expression on lung dendric cells, reduce lung *M. tuberculosis* burden, decrease regulatory T cells, and increase activated and effector memory CD4T cells exhibiting a CD44hi phenotype and a CD62LloCD44hi phenotype, respectively (45). This study indicated that the functions of lung dendric cells and T cell against *M. tuberculosis* in dysbiotic mice could be enhanced by probiotic *L. plantarum*. Although few studies showed the antagonistic and immunoregulatory effects against *M. tuberculosis*, these findings highlight the potential role of probiotics as a novel strategy in TB treatment.

The concept of postbiotics is proposed according to the findings that beneficial effects of bacteria are modulated by secreted metabolites. Postbiotics are inactivated microbial cells and/or their components that confer beneficial effects on host health (47). Microbial cell-wall fractions, extracellular or surface-associated proteinaceous molecules, exopolysaccharides, or microbial metabolic such as SCFA, vitamins, amino acids, peptides, etc, which could exert benefits to host health, directly or indirectly belong to postbiotics (48). Khusro et al. (49) purified and characterized an anti-tubercular protein produced by strain *Staphylococcus hominis* MANF2, with molecular mass 7712.3 Da. In addition, they found this inhibition effect was dose-dependent. Carroll et al. (50) found that lacticin 3147, an antimicrobial peptide produced by *Lactococcus lactis* subsp. *cremoris* MG1363, strongly inhibited the growth of *M. tuberculosis* H37Ra *in vitro*, with an MIC_90_ value of 7.5 mg/L, and demonstrated its greater potential as a therapeutic agent. Another antimicrobial protein produced by *Lactococcus lactis* subsp. *lactis* was also found to act against mycobacteria, which is associated with proton motive force collapse and intracellular ATP decrease (51). Indole propionic acid, a gut microbiota metabolite was also identified as an anti-tubercular agent (52–54). Negatu et al. (54) primarily screened 1,000 fragments in the Maybridge Ro3 library, and identified 29 compounds *in vivo* with the most anti-tubercular activity. Subsequently, 29 compounds were co-cultured with *M. tuberculosis* to determine their bactericidal activity against *M. tuberculosis*, and half of them could reduce *M. tuberculosis* viability 100-fold. Among these compounds, indole propionic acid showed the strongest inhibition effect against *M. tuberculosis*. Consequently, it was tested in a mouse model, which were infected with a low dose of *M. tuberculosis* by the aerosol route, and found to reduce bacterial load in spleen seven-fold, indicating its direct anti-tubercular activity. This research group further focused on the antibacterial mechanism of indole propionic acid. After metabolic, chemical rescue, genetic, and biochemical analyses, they found indole propionic acid could mimic physiological allosteric inhibitor of TrpE, block tryptophan biosynthesis in *M. tuberculosis*, and hence show antimycobacterial activity (53). These findings illustrate the potential anti-tubercular activity of postbiotics, although more research needs to be performed to elucidate microbiota and host factors involved in anti-tubercular activity, the role of postbiotics in TB susceptibility, progression and severity, and the application of postbiotics in anti-TB treatment.

## The Gut–Lung Axis in TB

Host systemic and lung immunity plays an important role in TB pathogenesis through controlling the clearance, survival, and replication of *M. tuberculosis* (55). Upon exposure to *M. tuberculosis*, alveolar epithelial cells are the first cell lines to recognize and bind to the outer surface molecules of mycobacteria through several types of PRRs, such as C-type lectins, and TLRs (56). Subsequently, several signaling pathways are activated to induce the secretion of cytokines and chemokines, and to initiate the migration of immune cells to the infection sites (55).

There are increasing numbers of studies illustrating the role of gut and pulmometry microbiota in modulating immune function in prevention, progression, and treatment of chronic respiratory diseases (57–61). The microbiota influences TB prevention, pathogenesis, and treatment mainly by affecting the percentage and function of immune cell subsets, producing bacteriocins and bacteriolysins that restrict the growth of *M. tuberculosis* directly, and/or by influencing bioavailability and pharmacokinetics of anti-TB drugs (62). Gut microbiota equilibrium has been shown to play an important role in regulating immune response through improving immune cell response against *M. tuberculosis* and promoting Th1/Th2 balance (31, 63, 64). Innate immune cells could be affected directly by gut microbiota and their metabolites, or indirectly by cytokines secreted by epithelium cells or dendritic cells, which in turn activate the migration of adaptive immune cells to infection sites (6). An altered gut microbial balance could lead to the suppressed ability of dendritic cells in antigen presentation, which consequently result in a diminished innate and adaptive immune response against *M. tuberculosis* (18). Expression of C-type lectins, a type of PRR would also be reduced by gut microbiota alterations, hence exerting an adverse effect on immune cell activation and *M. tuberculosis* clearance (45). In addition, gut microbiota alterations with increased abundance of *Bacteroides* and *Verrucomicrobiaceae*, and decreased abundance of *Lachnospiraceae* would result in a compromised anti-TB immune response with elevated numbers of T regulatory cells which increase susceptibility to TB, and decreased numbers of Th1 cells which promote protective immunity against *M. tuberculosis* (65). Gut microbiota metabolites could be produced and secreted into the bloodstream, and then transported to the lungs, thus stimulating the local immune response. Gut microbiota metabolites, such as butyrate and propionate, could decrease the lung production of IL-17, suppress Th1 immunity, and increase numbers of T regulatory cells, consequently influencing the outcome of *M. tuberculosis* infection (66–68). Another gut microbiota metabolite, indole propionic acid, could disturb tryptophan biosynthesis in *M. tuberculosis*, and hence inhibit its growth directly (53, 54). Lung microbiota also plays a key role in local immunity through affecting recruitment and activation of epithelial cells and T regulatory cells (69). The phylum Bacteroidetes has been shown to downregulate lung inflammatory status (70), whereas *Prevotella* spp. and *Veillonella* spp. could upregulate lung inflammatory status mediated by Type 17 helper T cells (71). In addition, respiratory commensal bacteria *Corynebacterium pseudodiphtheriticum* has been shown to improve the function of alveolar macrophages, and regulate the innate immune response against virus infection, indicating the potential role of *C. pseudodiphtheriticum* as a next-generation probiotic (72). There is a strong association between lung microbiota and gut microbiota; they overlap in microbiota composition, and microbiota diversity in two organs decrease or increase simultaneously (73). The alterations in gut microbiota could affect lung microbiota, which would influence lung inflammatory response and granuloma formation upon *M. tuberculosis* infection (10, 74). Meanwhile, the composition of lung microbiota also affects gut microbiota through translocation of microorganisms into blood (75). Therefore, the gastrointestinal tract and lung could influence the microbiota and immune function homeostasis of each other, and this bidirectional gut-lung axis consequently could influence the host immune response against *M. tuberculosis*. Novel supplementation, such as probiotics and postbiotics, could modulate microbiota and regulate immune function through competing with pathogenic bacteria, conferring antibacterial effects, regulating innate immune response, stimulating epithelial cell growth, and improving barrier function (76). Therefore, they could be applied as strategies in TB prevention and treatment.

## Conclusions

The gut-lung axis plays an important role in TB prevention and treatment outcome, through affecting host immune response against *M. tuberculosis*. Several studies have suggested that microbiota could play a significant role in TB pathogenesis and treatment efficiency, the dysbiosis of microbiota may result in adverse impacts on immune response to *M. tuberculosis* infection, a more serious development of granulomatous, and decreased efficiencies of anti-TB drugs. Meanwhile, multi-drugs used in TB treatment could significantly alter the gut and pulmonary microbiota community for a long time. Therefore, the microbiota becomes an inevitable subject in TB research area, and the identification and validation of microorganisms contributing to TB progression and treatment outcomes in epidemiologically representative populations should be undertaken. In addition, probiotics and postbiotics have exhibited anti-tuberculosis activity *in vitro* and *in vivo*, indicating their potential for application in anti-TB treatment to overcome complications caused by the current use of multiple antibiotics. Some members of respiratory commensal bacteria also show the potential to be used as next-generation probiotics in resistant respiratory infection. In summary, the microbiome will contribute to TB therapy efficiency, and the application of probiotics and postbiotics could be explored as an add-on to current therapies, or drug optimization strategies.

## Author Contributions

All authors listed have made a substantial, direct and intellectual contribution to the work, and approved it for publication.

## Conflict of Interest

The authors declare that the research was conducted in the absence of any commercial or financial relationships that could be construed as a potential conflict of interest.
